# POWER EXPANDS FUTURE TIME PERSPECTIVE AMONG OLDER ADULTS

**DOI:** 10.1093/geroni/igae098.2890

**Published:** 2024-12-31

**Authors:** Liru Wang, Mengxuan Su, Minjie Lu

**Affiliations:** Beijing Normal University at Zhuhai, Zhuhai, Guangdong, China (People’s Republic); Beijing Normal University at Zhuhai, Zhuhai, Guangdong, China (People’s Republic); Beijing Normal University at Zhuhai, Zhuhai, Guangdong, China (People’s Republic)

## Abstract

Social power has been conceptualized as the ability to control or influence others in meaningful ways (Fiske, 1993). According to Socioemotional Selectivity Theory (Carstensen, 2006), when aging, individuals tend to perceive their future as more limited. This project aims to examine whether older adults with power may perceive their future as more expanded. In Study 1, 241 participants aged from 18 to 80 (154 females, Mage=50.27, SD=16.76) reported their sense of their own power and perception of the future. Results showed that the sense of power was positively associated with an expanded future time perspective, measured by a future time perspective scale and a life-axis index. In Study 2, 55 older adults (24 females, Mage=64.25,SD=3.09) who are currently in authority at work were recruited for the high-power group, and 52 older adults (25 females, Mage=65.38,SD=3.68) without authority at word were recruited for the low-power group. Consistent with Study 1, the high-power group of older adults reported a more expanded future than did the low-power group. In addition, the high-power group of older adults displayed a stronger inclination to pursue knowledge-related goals (e.g., obtaining new skills and experiences, knowing new friends). In both studies, the effect of power on expanding future time perspective was mediated by the participants’ sense of control. These findings highlight that role of power in counteracting the age-related changes in future time perspective.

